# The Overlooked Factors: How Cardioplegia Delivery and Deairing May Shape Clinical Results

**DOI:** 10.1093/icvts/ivaf203

**Published:** 2025-08-19

**Authors:** Ignazio Condello

**Affiliations:** School of Medicine and Surgery, University of Insubria, Varese 21100, Italy

We read with great interest the article by Tauron-Ferrer and colleagues comparing Buckberg (BS) and Del Nido (DNS) cardioplegia solutions in patients undergoing isolated aortic valve replacement.^1^ We commend the authors for conducting a prospective, randomized, two-centre trial on this important topic in adult cardiac surgery. However, we would like to highlight some aspects that we believe deserve further exploration in future studies. In particular, there was significant heterogeneity in the route of cardioplegia administration between the groups: retrograde delivery and direct coronary ostia cannulation were notably more frequent in the BS group (51.7% and 49.7%, respectively) compared with the DNS group (0.69% and 15.3%). This variation may have substantially influenced myocardial protection outcomes and the electrophysiological behavior after aortic unclamping. Furthermore, contrary to what has been reported in other studies,^2^ no significant reduction in cardiopulmonary bypass (CPB) or cross-clamp times was observed in the DNS group compared to the BS group (CPB time 74 vs 78 minutes, *P* = .245; cross-clamp time 57.5 vs 58 minutes). This might be explained by the frequent use of retrograde cardioplegia in the BS group, which allowed a more continuous surgical workflow without interruptions for repeated antegrade dosing, offsetting the potential time-saving advantage typically attributed to Del Nido cardioplegia. Additionally, important perioperative management aspects such as deairing manoeuvres, lung ventilation strategies, cardiopulmonary bypass venting, and intraoperative CO_2_ management were not sufficiently detailed. These factors critically impact spontaneous rhythm recovery and ventricular fibrillation rates after cross-clamp removal. In this context, the role of transoesophageal echocardiography (TEE) in guiding and confirming optimal deairing should not be overlooked, as it is a fundamental tool to minimize air embolism and support stable myocardial reperfusion. While the study concludes that there are no significant differences between the two cardioplegia strategies regarding biochemical markers of myocardial injury (CK and Troponin T), we believe that stricter standardization of cardioplegia delivery routes, detailed reporting of deairing techniques, and TEE-guided management could reveal clinically meaningful differences that might otherwise remain hidden. We congratulate the authors once again for their valuable contribution and look forward to future studies that will further explore these important technical aspects to refine myocardial protection strategies in isolated valve surgery.

## Funding

None declared.

## Data Availability

There are no new data associated with this article.
